# From Melanocyte to Malignant Metastatic Melanoma

**DOI:** 10.1155/2010/798324

**Published:** 2010-09-19

**Authors:** Prashiela Manga, Keith S. Hoek, Lester M. Davids, Sancy A. Leachman

**Affiliations:** ^1^The Ronald O. Perelman Department of Dermatology, New York University School of Medicine, 522 First Avenue, New York, NY 10016, USA; ^2^Department of Dermatology, University Hospital Zurich, 8091 Zurich, Switzerland; ^3^Department of Human Biology, Faculty of Health Sciences, University of Cape Town, Cape Town 7925, South Africa; ^4^Huntsman Cancer Institute, University of Utah, Salt Lake City, UT 84112, USA

Incidence of melanoma, the deadliest form of skin cancer, continues to increase among older adults and young women worldwide despite significant efforts to raise awareness and inform the public about risk factors such as sun exposure, use of tanning salon, and the need to monitor skin for potential neoplastic lesions. Whether this increase is due to an actual rise in the number of individuals that develop the disease or due to an increase in classification of lesions as melanoma remains contentious; however it is indisputable that limited progress has been made in improving treatment of malignant melanoma or mortality rates. While new targeted agents against BRAF and immunotherapies directed against CTLA4 are showing great promise in clinical trials, FDA-approved therapies for melanoma remain largely ineffective, highlighting the need to better understand the mechanisms underlying disease initiation and progression. Melanoma results from malignant transformation of melanocytes. The most frequent site of transformation is in the skin where melanocytes produce the pigment melanin that confers skin color and protects against sun-induced damage. Multiple factors contribute to melanoma risk, including genetic predisposition and environmental risk factors such as sun exposure. In this special issue, we present six papers that review some of the crucial issues currently being investigated in the melanoma field.

B. Bandarchi et al. provide a broad review of malignant melanoma, from epidemiology and risk factors to classification and clinical features, highlighting some of the genes that play a role in disease progression. Furthermore they review treatment options and discuss the 2009 American Joint Committee on Cancer Melanoma Staging and Classification.

J. A. H. Lee explores the relationship between incidence rates for in situ and invasive melanoma, proposing that the trends may in fact provide clues to the mechanisms that underlie development and progression of melanoma.

R. Ria et al. review the events and consequences of increasing vascularization during tumorigenesis and summarize the most relevant cytokines involved. They also detail the roles of melanoma-produced factors involved in interacting with or modifying the extracellular environment in favour of neovascularizing processes. Finally, these authors discuss recent efforts to therapeutically target tumor vascularization in patients using a variety of small molecule inhibitors, concluding that there remains much room for improvement.

A group led by Annelies de Klein (T. van den Bosch et al.) compare cutaneous and uveal melanomas and find that while diagnosis and therapy options are similar, there are telling differences in the biology of these malignances. For example, cutaneous melanomas may metastasize to a variety of organs in the body while most uveal melanomas metastasize to the liver; the authors point out that this difference is likely due to the absence of lymphatic vessels associated with the eye. Perhaps most interesting are the cytogenetic differences discussed, the most prominent example being that monosomy 3 is a common feature of uveal melanomas which is rarer in the cutaneous disease.

J. F. G. Cohen-Solal and coworkers review melanoma's capacity for immune escape, reminding us of extensive studies conducted by themselves and other groups which show that expression of Fc-gamma receptor decoys serves to prevent immune effector cells from recognizing melanoma cells and providing answers as to how melanoma has so far avoided targeted immunotherapy. They close with the hopeful note that it may be possible to design Fc fragments with reduced affinity for decoys and higher affinity for immune-functional receptors.

The Italian group of M. Sanzo et al. review chronic stress as a possible cofactor in the progression of melanoma. As treatment of this dreaded disease continues to be ineffective, this hypothesis is worth exploring. They suggest in their paper that despite understanding biological mechanisms underlying local and distant metastases, the effect of stress mediator molecules such as catecholamines may increase progression. These findings point to the complexity of cancers in that chronic stress including both psychological and environmental factors may influence biological pathways. Finally, these authors suggest that intervention targeting these catecholinergic hormones in addition to psychological and social support may represent a valid approach in the treatment of advanced melanoma. 

*Prashiela Manga* *Prashiela Manga* *Keith S. Hoek* *Keith S. Hoek* *Lester M. Davids* *Lester M. Davids* *Sancy A. Leachman* *Sancy A. Leachman*

